# Alterations in Cancer Treatment During the First Year of the COVID-19 Pandemic in the US

**DOI:** 10.1001/jamanetworkopen.2023.40148

**Published:** 2023-10-30

**Authors:** Lauren M. Janczewski, Joseph Cotler, Ryan P. Merkow, Bryan Palis, Heidi Nelson, Timothy Mullett, Daniel J. Boffa

**Affiliations:** 1 Department of Surgery, Northwestern University Feinberg School of Medicine, Chicago, Illinois; 2 American College of Surgeons Cancer Programs, Chicago, Illinois; 3 Department of Surgery, The University of Chicago Pritzker School of Medicine, Chicago, Illinois; 4 Department of Surgery, University of Kentucky College of Medicine, Lexington; 5 Section of Thoracic Surgery, Department of Surgery, Yale School of Medicine, New Haven, Connecticut

## Abstract

**Importance:**

The COVID-19 pandemic created challenges to the evaluation and treatment of cancer, and abrupt resource diversion toward patients with COVID-19 put cancer treatment on hold for many patients. Previous reports have shown substantial declines in cancer screening and diagnoses in 2020; however, the extent to which the delivery of cancer care was altered remains unclear.

**Objective:**

To assess alterations in cancer treatment in the US during the first year of the COVID-19 pandemic.

**Design, Setting, and Participants:**

This retrospective cohort study used data from the National Cancer Database (NCDB) on patients older than 18 years with newly diagnosed cancer from January 1, 2018, to December 31, 2020.

**Main Outcomes and Measures:**

The main outcomes were accessibility (time to treatment, travel distance, and multi-institutional care), availability (proportional changes in cancer treatment between years), and utilization (reductions by treatment modality, hospital type) of cancer treatment in 2020 compared with 2018 to 2019. Autoregressive models forecasted expected findings for 2020 based on observations from prior years.

**Results:**

Of 1 229 654 patients identified in the NCDB in 2020, 1 074 225 were treated for cancer, representing a 16.8% reduction from what was expected. Patients were predominately female (53.8%), with a median age of 66 years (IQR, 57-74 years), similar to demographics in 2018 and 2019. Median time between diagnosis and treatment was 26 days (IQR, 0-36 days) in 2020, and median travel distance for care was 11.1 miles (IQR, 5.0-25.3 miles), similar to 2018 and 2019. In 2020, fewer patients traveled longer distances (20.2% reduction of patients traveling >35 miles). The proportions of patients treated with chemotherapy (32.0%), radiation (29.5%), and surgery (57.1%) were similar to those in 2018 and 2019. Overall, 146 805 fewer patients than expected underwent surgery, 80 480 fewer received radiation, and 68 014 fewer received chemotherapy. Academic hospitals experienced the greatest reduction in cancer surgery and treatment, with a decrease of approximately 484 patients (−19.0%) per hospital compared with 99 patients (−12.6%) at community hospitals and 110 patients (−12.8%) at integrated networks.

**Conclusions and Relevance:**

This study found that among patients diagnosed with cancer in 2020, access and availability of treatment remained intact; however, reductions in treated patients varied across treatment modalities and were greater at academic hospitals than at community hospitals and integrated networks compared with expected values. These results suggest the resilience of cancer service lines and frame the economic losses from reductions in cancer treatment during the pandemic.

## Key Points

**Question:**

Was the delivery of cancer treatment across all cancer sites altered during the first year of the COVID-19 pandemic in the US?

**Findings:**

In this cohort study of 1 229 654 patients identified in the National Cancer Database in 2020, 1 074 225 patients received cancer treatment, representing a 16.8% reduction from 2018 to 2019. Although cancer treatments were both accessible and available for patients who were able to be diagnosed, reductions in cancer surgery and treatment at academic hospitals, compared with community hospitals and integrated networks, were greatest.

**Meaning:**

This study showed the resilience of cancer service lines during the COVID-19 pandemic.

## Teaser

This cohort study assesses alterations in cancer treatment during the first year of the COVID-19 pandemic among adults in the US.

## Introduction

The first year of the COVID-19 pandemic created unprecedented strains on the US health care system, including the delivery of cancer care.^1^ Abrupt resource diversion in 2020 toward patients with COVID-19 and the uncertainty over the hazards of SARS-CoV-2 infection for patients and health care workers put cancer evaluation and treatment on hold for many patients.^2,3^ Previous reports have shown substantial declines in the use of cancer screening in 2020 as well as routine primary care evaluations, which are commonly the inroads to cancer diagnosis.^4,5^ As a result, it has been estimated that 206 099 fewer patients than expected were diagnosed with cancer in 2020.^6^

The same challenges that prevented patients from being diagnosed with cancer in 2020 may have also impaired cancer treatment. For many hospitals, the space, personnel, and facilities normally involved in cancer treatment overlapped with those needed to accommodate the population with COVID-19.^7,8^ Furthermore, real or anticipated supply chain challenges, early impressions of the hazards of contracting COVID-19, and projections of the duration of the pandemic’s impact led many organizations to propose guidance toward delaying or altering cancer treatment.^9,10^ However, the extent to which the delivery of cancer care was altered in the first year of the pandemic is unclear.

The National Cancer Database (NCDB) captures data from roughly 75% of patients newly diagnosed with cancer in the US each year.^11^ We examined patterns of cancer treatment in 2020 and compared these with what was expected based on observations from the pre–COVID-19 era. Our objectives were to evaluate the accessibility and availability of cancer treatments in 2020 as a reflection of the response of cancer operations within the US health care system. We also examined patterns in the reductions of provided treatment to better characterize the consequences of the first year of the pandemic on different cancer service lines and hospital types.

## Methods

### Data Source

The NCDB is jointly maintained by the American College of Surgeons Commission on Cancer (CoC) and the American Cancer Society, representing 1 of the largest and most detailed tumor registries worldwide. The database tracks both the diagnosis and the treatment of cancer at CoC-accredited hospitals as an obligatory component of accreditation.^11,12^ Previous work^13^ evaluated the completeness of data submission for cancers diagnosed in 2020, examining whether the decline in captured data was associated with decreases in diagnoses made or with an impaired data collection infrastructure. That work concluded that the data collection infrastructure was maintained during the COVID-19 pandemic, supporting the use of 2020 NCDB data as a reflection of US cancer operations. This cohort study was conducted in compliance with the institutional review board protocol of the American College of Surgeons, with informed consent requirements waived because the data were deidentified. The Strengthening the Reporting of Observational Studies in Epidemiology (STROBE) reporting guideline for cohort studies was followed.

### Patient Population

The NCDB was queried for patients older than 18 years with newly diagnosed cancer and treated at CoC-accredited programs from January 1, 2018, through December 31, 2020. The analysis included all 70 cancer sites collected by the NCDB (eg, lung, colon, and breast), identifying patients who received cancer treatment (eFigure 1 in Supplement 1).

### Independent Variables

The following variables were examined across the patient population: age at diagnosis (years), race and ethnicity, sex, time from diagnosis to treatment (days), travel distance (miles), treatment in more than 1 CoC facility, treatment modality (surgery, chemotherapy, and radiation), and facility type.^14^ Race and ethnicity were self-reported and categorized as Asian, Native Hawaiian, or Other Pacific Islander; Hispanic; non-Hispanic Black; non-Hispanic White; and other or unknown (defined by the lack of documentation of the patient’s race and ethnicity according to the NCDB dictionary).

### Primary Outcomes

The primary outcomes of this study concentrated on 3 broad perspectives of cancer care during the COVID-19 pandemic: accessibility, availability, and utilization. These outcomes were extrapolated from collected data.

#### Accessibility of Treatment

Accessibility of treatment during the pandemic was examined through 3 different treatment perspectives and was compared with the forecasted, or expected, data based on observations from 2018 to 2019. First, the time from cancer diagnosis to treatment initiation was evaluated across all modalities (time to treatment). Next, the distance traveled to receive care was evaluated (travel distance), defined as the distance in miles between the patient’s residence and the hospital reporting the case for which care was received. Lastly, the proportion of patients receiving treatment from multiple institutions was examined, as coordinating multi-institutional care would have been more challenging during the first year of the pandemic.

#### Availability of Treatment

Availability of treatment was assessed by examining the proportion of patients who received surgery, chemotherapy, and radiation. Reductions in treatment availability would presumably result in smaller proportions of patients being treated with that modality.

#### Utilization of Treatment

Differences between the observed number of treated patients and the expected number were used to estimate reductions in provided care during 2020. These analyses were performed across different treatment modalities (surgery, chemotherapy, and radiation) and hospital types (academic hospitals, community hospitals, and integrated networks). The NCDB considers academic hospitals to be facilities that participate in postgraduate medical education with more than 500 new cancer cases annually, community hospitals to be those that do not participate in postgraduate medical education, and integrated networks to be facilities belonging to an integrated organization overseen by a centralized governance.^14^

### Statistical Analysis

For accessibility, trends in time-to-treatment analyses for surgery, chemotherapy, and radiation from 2018 through 2020 were evaluated using bayesian analysis procedures in R, version 4.3.1 (R Foundation for Statistical Computing). Independent autoregressive time-series models were developed using monthly observations between January 1, 2018, and December 31, 2019 (24 data time points) as input into a regression equation, estimating expected values for age at diagnosis, travel distance, muti-institutional care, and treatment utilization by modality (surgery, chemotherapy, and radiation) and facility type (academic hospital, community hospital, and integrated network) in 2020 using SAS, version 9.4 (SAS Institute Inc). Models were adjusted for monthly and seasonal trends, which may impact the timing of reported cancer diagnoses, and were set at a 90% credible interval. Model precision assessments were not performed, as we knew that the estimated values in 2020 would be incorrect given pandemic-related changes to the health care system. Rather, our outcome for this study examined differences between the generated expected values for 2020 and what was truly observed. Descriptive univariate statistics using χ^2^ tests were performed to compare observed vs expected treatment findings. Two-sided *P* < .05 using Bonferroni correction was considered significant.

Because different cancers have distinct treatment requirements and may have been impacted in different ways during the pandemic, sensitivity analyses were performed examining 5 specific tumor sites. Patients diagnosed with tumors of the breast, colon, lung, melanoma, and stomach were studied as separate cohorts. These were chosen to represent different oncologic treatment teams, as the patients may have had different impressions or experiences of cancer care during this time.

## Results

### Patients

Of 1 229 654 patients identified in the NCDB in 2020, 155 429 (12.6%) did not receive cancer treatment and were excluded from the treatment analysis. A total of 1 074 225 patients were treated for cancer in 2020, representing a 16.8% reduction from what was expected. The median age of treated patients was 66 years (IQR, 57-74 years). The greatest reduction in treated patients by age group was among those aged 90 years or older (−35.5%) (eTable 1 in Supplement 1). Patients were predominately female (661 872 [53.8%]) and non-Hispanic White (926 984 [75.4%]), similar to demographics in the 2 prior years (Table). A total of 567 151 (46.1%) were male; 43 033 (3.5%), Asian, Native Hawaiian, or Other Pacific Islander; 81 955 (6.7%), Hispanic; 135 555 (11.0%), non-Hispanic Black; and 42 127 (3.4%), other or unknown race and ethnicity. The most common cancer sites were breast (233 331 [19.0%]), lung (140 355 [11.4%]), and prostate (124 822 [10.2%]), also similar to prior years.

**Table.** Patient Demographics in 2018, 2019, and 2020

Characteristic	Patients		
	2018 (n = 1 400 657)	2019 (n = 1 437 754)	2020 (n = 1 229 654)
Age, median (IQR), y	66 (57-74)	66 (57-74)	66 (57-74)
Sex, No. (%)			
Female	753 831 (53.8)	770 524 (53.6)	661 872 (53.8)
Male	646 371 (46.1)	666 779 (46.4)	567 151 (46.1)
Unknown	455 (0.1)	451 (<0.1)	631 (0.1)
Race and ethnicity, No. (%)			
Asian, Native Hawaiian, or Other Pacific Islander	48 551 (3.5)	50 900 (3.5)	43 033 (3.5)
Hispanic	91 936 (6.6)	96 178 (6.7)	81 955 (6.7)
Non-Hispanic Black	153 693 (11.0)	158 773 (11.0)	135 555 (11.0)
Non-Hispanic White	1 060 226 (75.7)	1 085 244 (75.5)	926 984 (75.4)
Other or unknown^a^	46 251 (3.3)	46 659 (3.3)	42 127 (3.4)
Most common disease sites, No. (%)			
Breast	260 284 (18.6)	268 193 (18.7)	233 331 (19.0)
Lung	163 342 (11.7)	165 597 (11.5)	140 355 (11.4)
Prostate	144 490 (10.3)	153 497 (10.7)	124 822 (10.2)
Urinary system	110 174 (7.9)	113 733 (7.9)	98 552 (8.0)
Female genital system	88 191 (6.3)	89 559 (6.2)	78 250 (6.4)
Travel distance, median (IQR), mi	11.1 (4.9-25.7)	11.1 (5.0-25.7)	11.1 (5.0-25.3)
Time to treatment, median (IQR), d			
Overall	27 (0-36)	27 (0-37)	26 (0-36)
Surgical	26 (1-53)	27 (1-55)	26 (0-53)
Chemotherapy	42 (21-56)	42 (22-56)	40 (21-54)
Radiation	84 (41-103)	85 (42-105)	83 (41-104)

^a^ Other or unknown, according to the National Cancer Database data dictionary, was defined by the lack of documentation of the patient’s race or ethnicity. Because race or ethnicity was not stated in the patient’s record, it was coded as other or unknown by a certified tumor registrar for data abstraction.

### Accessibility of Treatment

**Figure 1.**
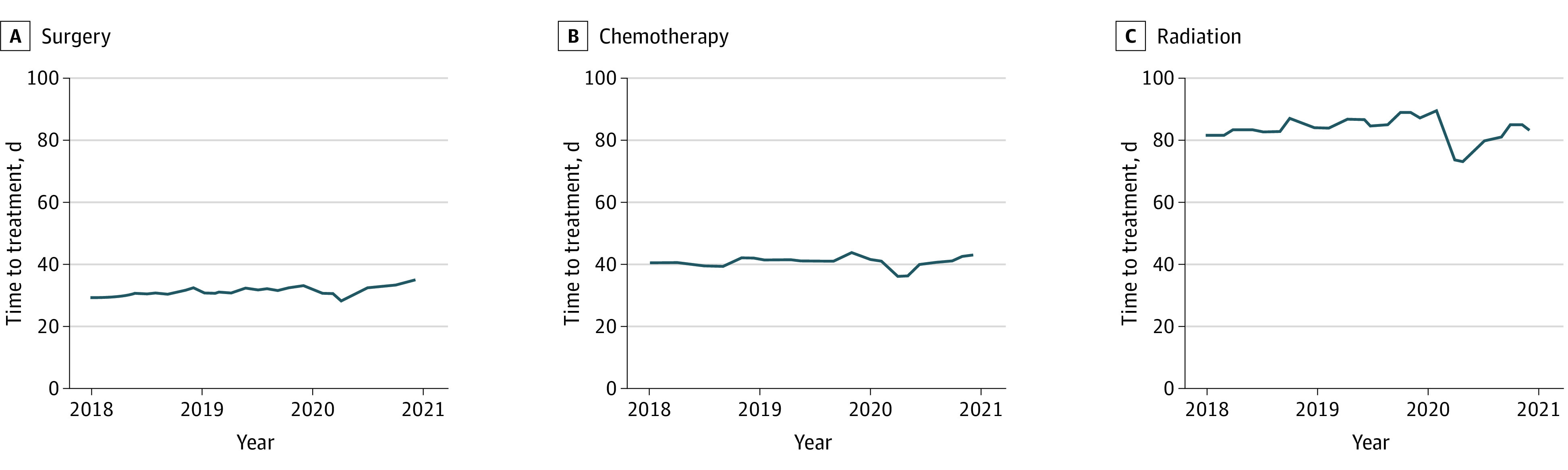
Trend in Time From Diagnosis to Treatment Initiation From 2018 Through 2020

The median time between diagnosis and treatment initiation in 2020 was 26 days (IQR, 0-36 days), which was similar to the median time in 2018 and 2019 (Table). Time to treat was examined over the study period (Figure 1). Prior to the COVID-19 pandemic, monthly variation was observed, with increased treatment time toward the end of each calendar year. In 2020, there was a noticeable reduction in the time to treatment initiation beginning in March and April seen across all modalities. However, within 3 months, treatment times returned to pre–COVID-19 pandemic durations.

Travel distance was examined as a separate parameter of accessibility. In 2020, patients traveled a median of 11.1 miles (IQR, 5.0-25.3 miles), similar to prior years (Table). However, there was a greater-than-expected decrease in patients traveling farther distances. For example, the subset of patients traveling more than 35 miles experienced a larger decline (−20.2%) compared with patients who were cared for between 5 miles and 35 miles (5-10 miles: −15.8%; 10-15 miles: −16.3%; and 15-35 miles: −15.7%) (eTable 2 in Supplement 1). Additionally, travel distance and hospital type were highly correlated; patients at academic hospitals more often traveled more than 35 miles to receive care (27.1%) compared with those at integrated networks (11.8%) and community hospitals (12.9%) (Cramer *V*, 0.14; *P* < .001).

Finally, the prevalence of receiving treatment from more than 1 facility was examined. Overall, the population of patients receiving multi-institutional care had a greater decline (−17.0%) in 2020 compared with single institutional care (−14.5%) (eTable 3 in Supplement 1).

### Availability of Treatment

**Figure 2.**
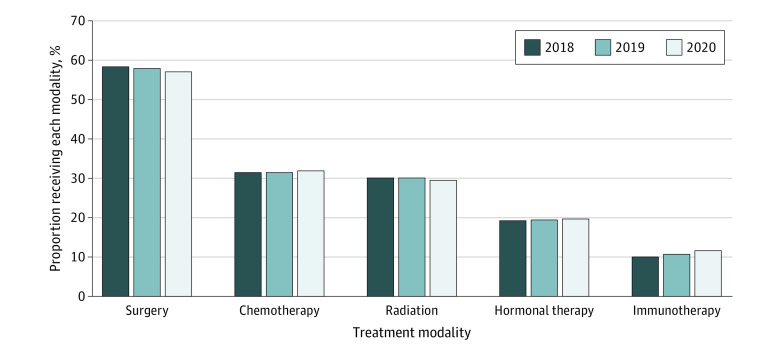
Proportion of Patients Receiving Each Treatment Modality During the COVID-19 Pandemic

The proportion of patients receiving chemotherapy, radiation, or surgery was evaluated in all 3 years (Figure 2). While the percentage of patients receiving chemotherapy (32.0% in 2020 vs 31.4% in 2019 and 31.5% in 2018) and radiation (29.5% in 2020 vs 30.1% in 2019 and 30.2% in 2018) remained largely stable over time, there was a slight decline in the proportion of patients receiving surgery in 2020 (57.1% vs 58.1% in 2019 and 58.6% in 2018). The proportion of patients who were excluded because they did not receive any treatment in 2020 remained similar as well (12.6% vs 12.4% in 2019 and 12.2% in 2018).

### Utilization of Treatment

**Figure 3.**
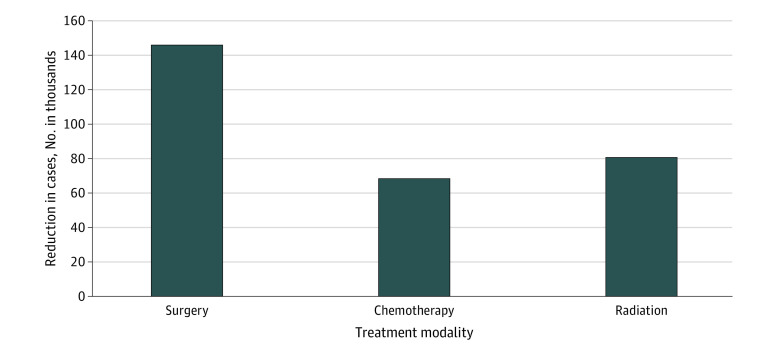
Reductions in Treatment Utilization in 2020 Compared With 2018 and 2019 For all comparisons, there was a significant change at *P* < .05.

To understand the consequences of the pandemic on cancer service lines, patterns of reductions in treated patients were examined across treatment types and hospitals. Overall, there were 146 805 (−17.3%) fewer than expected surgically treated patients, 68 014 (−14.8%) fewer receiving chemotherapy, and 80 480 (−18.2%) fewer treated with radiation (Figure 3 and eTable 4 in Supplement 1).

**Figure 4.**
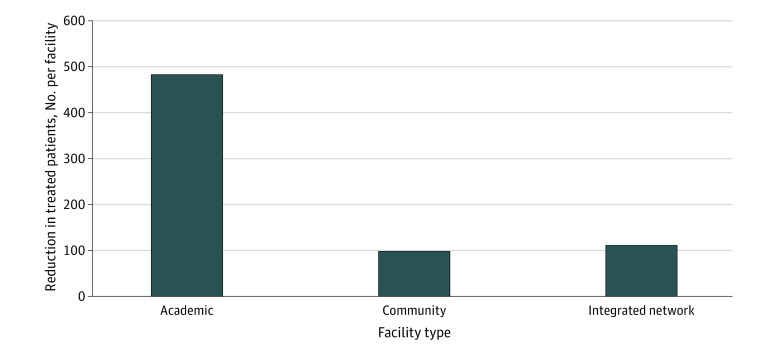
Approximate Declines in Treated Patients by Facility Type During the COVID-19 Pandemic

Variance in cancer treatment across different facility types was also assessed as a parameter of utilization. Academic facilities experienced greater reductions in treated patients in 2020 (−484 [−19.0%] per hospital) compared with community hospitals (−99 [−12.6%] per hospital) and integrated networks (−110 [−12.8%] per hospital) (Figure 4 and eTable 5 in Supplement 1). The number of academic hospitals represented the smallest group compared with community hospitals and integrated networks, which amplified the relative reduction (eFigure 2 in Supplement 1).

### Sensitivity Analyses

Because of the potential for important nuances to differ by cancer site, several of the analyses were repeated across 5 specific cancer sites (breast, colon, lung, melanoma, and stomach). Overall, the patterns within the 5 cancer types mirrored observations of the larger cohort (eTables 2-5 and eFigure 3 in Supplement 1). However, there was some variability between tumor types. For accessibility, there were greater reductions in treated patients with melanoma traveling shorter distances (−34.3% among patients traveling <5 miles). In terms of treatment modalities, surgical treatment had the greatest decline for most cancers, but for lung cancer, the greatest reductions were in radiotherapy.

## Discussion

For patients with cancer who were able to be evaluated in the first year of the pandemic, patterns in treatment access and availability appeared to mimic the 2 preceding years. Specifically, time to treatment and travel distance were similar to those in 2018 and 2019. This is contradictory to most literature surrounding time to treatment reported during the pandemic. Prior reports frequently described challenges in delivering cancer care during this time, resulting in treatment delays, which negatively impacted the quality of care.^2,15,16^ However, in the current study, time to treatment initiation was maintained. This may reflect the overall perception of oncologists at the time—that patients had to be treated quickly before pandemic-related restrictions intensified.^17^ Alternatively, this could indicate fewer patients seeking treatment, as fewer patients were diagnosed in 2020^18,19^ and prior work from the NCDB suggested a 14.4% reduction in newly diagnosed cancer cases.^6^ However, most reports^2,15,18^ citing treatment delays during the first year of the COVID-19 pandemic were from smaller sample populations and may not have been representative of the experience of cancer treatment across the US. The current study is 1 of the largest, most comprehensive evaluations of cancer treatment during the pandemic to date, which may potentially explain these differences in findings. Additionally, it appeared that fewer patients traveled longer distances for care, and a smaller proportion of patients received multi-institutional care. This likely represents the emergence of stay-at-home orders and travel restrictions, as patients either wanting or needing to travel for specialized or coordinated cancer treatment were unable to do so.^20,21^

Treatment availability appeared to have remained relatively intact in 2020, as the proportion of patients receiving each type of treatment was similar to that in 2018 and 2019. The stability in use of all treatment modalities contradicted prior studies that reported significant reductions in the use of chemotherapy and cancer surgeries in 2020.^2,22,23^ Several factors could have influenced treatment availability. On the one hand, the creation of several consensus statements for guidance on appropriate triage of patients requiring cancer operations would suggest that a greater decline in operative volume should have been demonstrated.^24,25^ Additionally, 2020 was a time when elective and semi-elective procedures were deferred in many institutions due to a lack of resources, including personal protective equipment, and to create capacity to care for patients with COVID-19.^1,9^ On the other hand, this unanticipated stability in treatment availability may be attributed to the increase in global communication at the time. The medical field across all specialties, including cancer care, became a tighter community with efforts made from health care workers worldwide to prevent lapses in cancer treatment. For example, a COVID-19 minimal surgical pathway developed to standardize best practices in preventing surgical patients from contracting COVID-19 demonstrated that it was possible to safely perform surgical procedures during that time.^26^ This is 1 example among many that may collectively explain how patients with cancer continued receiving treatment as a result of the advocacy of health care workers.^27,28,29^

The reductions in treated patients (−16.8%) were high and appeared to exceed reductions in patients with diagnosed cancer (−14.4%),^6^ as a total of 155 429 patients in 2020 did not receive any form of treatment. The concern is that such a large number of untreated cancers would result in compromised outcomes for these patients over the next several years, and follow-up studies will be crucial in answering this important question.

Reductions in provided care were not uniformly distributed across treatment modalities or hospitals. The reduction in the number of patients receiving surgery was nearly double the reductions in patients receiving chemotherapy or radiation, and academic hospitals appeared to be particularly affected. As prior work has demonstrated a lack of stage migration during the pandemic,^6^ it is unlikely that the reduction in cancer surgery was attributable to an increase in patients presenting with advanced stage disease. It is tempting to try to extrapolate reductions in provided cancer care to understand the havoc that the pandemic brought to the health care economy.^30,31,32^ There is no way to generalize the impact of the pandemic from any perspective, let alone the complex and highly nuanced arena of hospital finances. That being said, there is at least an opportunity to scale the economic influence on cancer service lines. Although variable by disease site and clinical scenario, if we assess approximate crude costs for surgical treatment, chemotherapy, and radiation treatment of cancer, the lost revenue for US cancer service lines would equate to hundreds of millions of dollars.^33,34^ However, this figure ignores lost revenue from the multitude of supporting services, such as imaging, testing, and facility fees for treatment. The true losses from reductions in cancer treatment were likely orders of magnitude larger.

It is also worth a cautious examination of the relative changes across the health care economy (again recognizing profound simplifications). Surgical treatment, which is a major contributor to the operating revenue of many hospitals, appeared to be particularly impacted. There also was a signal that cancer service lines at academic hospitals were particularly impacted, as academic hospitals experienced nearly 4 times the reductions in provided care. This may be a contributing factor to the economic hardship reported by many academic health systems across the country as a result of resource diversion from academic medical centers during the COVID-19 pandemic given that patients with severe illness due to infection with SARS-CoV-2 were escalated to tertiary care centers early in the pandemic.^35,36^ This is not meant to imply that community hospitals have not been devastated by the pandemic. Rather, this is an attempt to tease out the cancer component of the financial disruption, recognizing that the magnitude of reductions may be exaggerated because academic hospitals tend to be larger.

The impact of the pandemic in 2020 was experienced differently at different times in the US as the surge progressed across the country from the coasts inward.^37^ Therefore, overall treatment disruptions throughout the year may not be reflective of the situation in any 1 location at any 1 time. This high-level perspective is not an attempt to perform a deep dive into disease-specific changes associated with the pandemic. Examining the year in total by all cancer sites allowed a general, overall perspective to be evaluated that included the maximal strain experienced by cancer service lines and the efforts to restore them.

### Limitations

There are several limitations to this study in addition to those typically ascribed to observational studies. First, the NCDB only includes CoC hospitals, and thus, these results may not be generalizable to the experience at non-CoC hospitals during the pandemic. Second, any study involving the first year of the pandemic must reconcile the challenge of the data collection infrastructure, which could have been impacted. Although prior work suggests that the data collection infrastructure remained intact,^13^ it is important to note that the US was affected in different ways at different times during the pandemic, possibly explaining why anticipated changes were not reflected in these high-level data. Third, interpretation of reductions in cancer treatment must also be done in the context of reductions in cancer diagnoses, of which prior work estimated more than 200 000 fewer cases during the first year of the pandemic.^6^

## Conclusions

In this cohort study analyzing data of US patients treated for any type of malignant tumor from 2018 to 2020, it appeared that access and availability of cancer treatment remained largely intact during the first year of the pandemic, suggesting a fair degree of resilience to cancer treatment in the US. The changes in utilization were heterogenous, with variable reductions in treated patients across different treatment modalities and greater reductions at academic hospitals.

## Notes

Supplement 1. **eFigure 1.** Consort Diagram **eTable 1.** Changes in Age of Treated Patients by Cancer Site in the Year 2020 **eTable 2.** Travel Distance in Miles by Cancer Site in the Year 2020 **eTable 3.** Coordination of Care Received at Single vs Multiple Institutions by Cancer Site in the Year 2020 **eTable 4.** Utilization of Treatment in the Year 2020 Compared With Prior Years **eTable 5.** Utilization of Treatment by Facility Type During the COVID-19 Pandemic **eFigure 2.** Number of Hospitals per Facility Type in the NCDB **eFigure 3.** Utilization of Treatment in the Year 2020 Compared With Prior Years by Primary Site

Supplement 2.
Data Sharing Statement
