# Supplementary material for: Alterations in Cancer Treatment During the First Year of the COVID-19 Pandemic in the US
Source: JAMA Netw Open. 2023 Oct 30;6(10):e2340148. doi: 10.1001/jamanetworkopen.2023.40148 (PMC10616721; doi:10.1001/jamanetworkopen.2023.40148)
Supplement: Supplement 1. — eFigure 1. Consort Diagram eTable 1. Changes in Age of Treated Patients by Cancer Site in the Year 2020 eTable 2. Travel Distance in Miles by Cancer Site in the Year 2020 eTable 3. Coordination of Care Received at Single vs Multiple Institutions by Cancer Site in the Year 2020 eTable 4. Utilization of Treatment in the Year 2020 Compared With Prior Years eTable 5. Utilization of Treatment by Facility Type During the COVID-19 Pandemic eFigure 2. Number of Hospitals per Facility Type in the NCDB eFigure 3. Utilization of Treatment in the Year 2020 Compared With Prior Years by Primary Site [file jamanetwopen-e2340148-s001.pdf]

Supplemental Online Content

Janczewski LM, Cotler J, Merkow RP, et al. Alterations in cancer treatment during the first year of the COVID-19 pandemic in the US. *JAMA Netw Open*. 2023;6(10):e2340148. doi:10.1001/jamanetworkopen.2023.40148

eFigure 1. Consort Diagram
eTable 1. Changes in Age of Treated Patients by Cancer Site in the Year 2020
eTable 2. Travel Distance in Miles by Cancer Site in the Year 2020
eTable 3. Coordination of Care Received at Single Versus Multiple Institutions by Cancer Site in the Year 2020
eTable 4. Utilization of Treatment in the Year 2020 Compared With Prior Years
eTable 5. Utilization of Treatment by Facility Type During the COVID-19 Pandemic
eFigure 2. Number of Hospitals per Facility Type in the NCDB
eFigure 3. Utilization of Treatment in the Year 2020 Compared With Prior Years by Primary Site

This supplemental material has been provided by the authors to give readers additional information about their work.

© 2023 Janczewski LM et al. *JAMA Network Open*.

eFigure 1. Consort Diagram

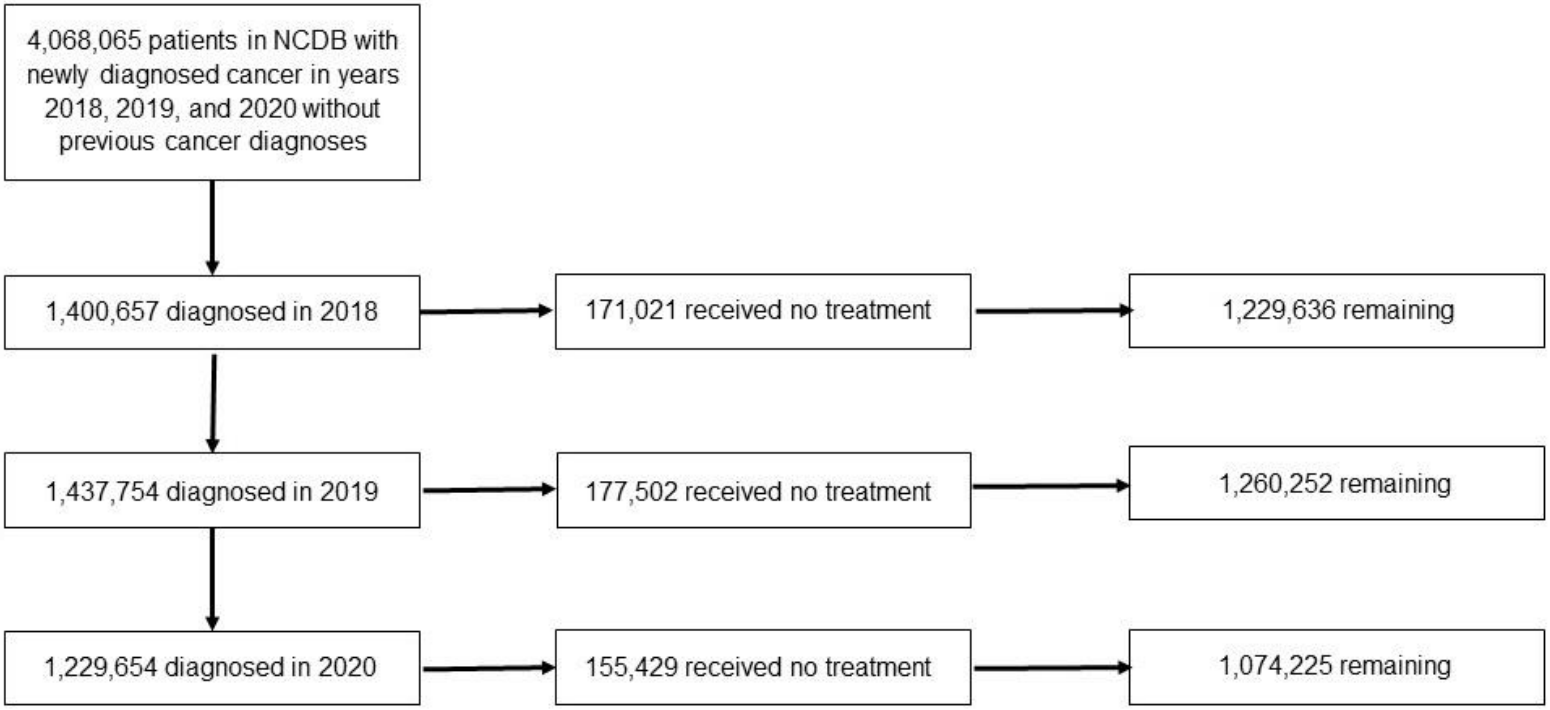

© 2023 Janczewski LM et al. *JAMA Network Open*.

eTable 1. Changes in Age of Treated Patients by Cancer Site in the Year 2020

Primary Site	Age at Diagnosis (years)	Observed No. (%)	Expected No. (% [95% CI])	Corrected p-Value	Observed - Expected	% Decrease in 2020
All Sites Combined	18 - 39	61,728 (5.0)	79,615 (5.4 [5.3,5.4])	<.001	-17887	22.47
	40 - 49	96,112 (7.8)	121,249 (8.2 [8.1,8.2])	<.001	-25137	20.73
	50 - 59	214,985 (17.5)	267,842 (18.1 [18.0,18.2])	<.001	-52857	19.73
	60 - 69	370,356 (30.1)	434,876 (29.4 [29.3,29.5])	<.001	-64520	14.84
	70 - 79	324,119 (26.4)	371,699 (25.1 [25.0,25.2])	<.001	-47580	12.80
	80 - 89	139,168 (11.3)	168,686 (11.4 [11.3,11.4])	0.03	-29518	17.50
	90 +	23,186 (1.9)	35,970 (2.4 [2.4,2.5])	<.001	-12784	35.54
Breast	18 - 39	10,812 (4.6)	13,063 (4.7 [4.7,4.8])	0.09	-2251	17.23
	40 - 49	32,857 (14.1)	38,953 (14.1 [14.0,14.3])	>.99	-6096	15.65
	50 - 59	51,070 (21.9)	60,448 (21.9 [21.8,22.1])	>.99	-9378	15.51
	60 - 69	66,897 (28.7)	77,875 (28.3 [28.1,28.4])	<.001	-10978	14.10
	70 - 79	51,707 (22.2)	58,854 (21.4 [21.2,21.5])	<.001	-7147	12.14
	80 - 89	17,333 (7.4)	21,485 (7.8 [7.7,7.9])	<.001	-4152	19.33
	90 +	2,655 (1.1)	4,749 (1.7 [1.7,1.8])	<.001	-2094	44.10
Colon	18 - 39	3,137 (3.6)	3,396 (3.3 [3.1,3.4])	<.001	-259	7.63
	40 - 49	7,952 (9.0)	8,768 (8.4 [8.2,8.6])	<.001	-816	9.31
	50 - 59	17,349 (19.7)	21,122 (20.2 [20.0,20.5])	<.001	-3773	17.86
	60 - 69	22,881 (26.0)	27,423 (26.3 [26.0,26.5])	0.43	-4542	16.56
	70 - 79	20,653 (23.5)	24,505 (23.5 [23.2,23.7])	>.99	-3852	15.72
	80 - 89	13,172 (15.0)	15,684 (15.0 [14.8,15.2])	>.99	-2512	16.02
	90 +	2,852 (3.2)	3,448 (3.3 [3.2,3.4])	>.99	-596	17.27
Lung	18 - 39	779 (0.5)	1,405 (0.8 [0.8,0.8])	<.001	-626	44.54
	40 - 49	3,204 (2.2)	4,391 (2.5 [2.4,2.6])	<.001	-1187	27.02
	50 - 59	18,621 (12.7)	23,654 (13.6 [13.4,13.7])	<.001	-5033	21.28
	60 - 69	48,389 (33.1)	55,068 (31.6 [31.4,31.9])	<.001	-6679	12.13
	70 - 79	51,207 (35.0)	59,889 (34.4 [34.2,34.6])	<.001	-8682	14.50
	80 - 89	21,461 (14.7)	26,152 (15.0 [14.9,15.2])	0.002	-4691	17.94
	90 +	2,454 (1.7)	3,519 (2.0 [2.0,2.1])	<.001	-1065	30.27
Melanoma	18 - 39	3,643 (7.0)	5,166 (7.6 [7.4,7.8])	<.001	-1523	29.48
	40 - 49	4,438 (8.6)	6,043 (8.9 [8.7,9.1])	0.07	-1605	26.56
	50 - 59	8,397 (16.2)	11,455 (16.8 [16.6,17.1])	<.001	-3058	26.70

© 2023 Janczewski LM et al. *JAMA Network Open*.

	**60 - 69**	14,025 (27.1)	17,759 (26.1 [25.8,26.4])	<.001	-3734	21.03
	**70 - 79**	13,542 (26.1)	16,769 (24.7 [24.3,25.0])	<.001	-3227	19.24
	**80 - 89**	6,621 (12.8)	8,708 (12.8 [12.6,13.1])	>.99	-2087	23.97
	**90 +**	1,166 (2.2)	2,098 (3.1 [3.0,3.2])	<.001	-932	44.44
**Stomach**	**18 - 39**	547 (3.4)	676 (3.5 [3.3,3.8])	>.99	-129	19.03
	**40 - 49**	1,133 (7.0)	1,396 (7.3 [6.9,7.7])	0.75	-263	18.82
	**50 - 59**	2,625 (16.2)	3,150 (16.5 [16.0,17.0])	>.99	-525	16.67
	**60 - 69**	4,609 (28.4)	5,346 (28.0 [27.4,28.6])	>.99	-737	13.79
	**70 - 79**	4,497 (27.7)	5,153 (27.0 [26.4,27.6])	0.27	-656	12.72
	**80 - 89**	2,357 (14.5)	2,808 (14.7 [14.2,15.2])	>.99	-451	16.04
	**90 +**	460 (2.8)	562 (2.9 [2.7,3.2])	>.99	-102	18.09

© 2023 Janczewski LM et al. *JAMA Network Open*.

eTable 2. Travel Distance in Miles by Cancer Site in the Year 2020

Primary Site	Travel Distance	Observed No. (%)	Expected No. (% [95% CI])	Corrected p-Value	Observed - Expected	% Decrease in 2020
All Sites Combined	**Within 5 miles**	308,030 (25.1)	371,244 (25.1 [25.0,25.1])	>.99	-63214	17.03
	**5-10 miles**	263,786 (21.5)	313,359 (21.2 [21.1,21.2])	<.001	-49573	15.82
	**10-15 miles**	170,048 (13.8)	203,254 (13.7 [13.7,13.8])	0.003	-33206	16.34
	**15-35 miles**	271,432 (22.1)	322,145 (21.7 [21.7,21.8])	<.001	-50713	15.74
	**> 35 miles**	216,358 (17.6)	271,247 (18.3 [18.2,18.4])	<.001	-54889	20.24
Breast	**Within 5 miles**	63,667 (27.3)	75,954 (27.5 [27.4,27.7])	0.06	-12287	16.18
	**5-10 miles**	56,376 (24.2)	66,172 (24.0 [23.8,24.1])	0.17	-9796	14.80
	**10-15 miles**	35,511 (15.2)	41,974 (15.2 [15.1,15.3])	>.99	-6463	15.40
	**15-35 miles**	49,861 (21.4)	57,385 (20.8 [20.6,20.9])	<.001	-7524	13.11
	**> 35 miles**	27,916 (12.0)	34,531 (12.5 [12.4,12.6])	<.001	-6615	19.16
Colon	**Within 5 miles**	25,318 (28.8)	30,184 (28.9 [28.6,29.2])	>.99	-4866	16.12
	**5-10 miles**	20,148 (22.9)	23,565 (22.6 [22.3,22.8])	0.12	-3417	14.5
	**10-15 miles**	11,873 (13.5)	13,857 (13.3 [13.1,13.5])	0.3	-1984	14.32
	**15-35 miles**	17,828 (20.3)	21,130 (20.2 [20.0,20.5])	>.99	-3302	15.63
	**> 35 miles**	12,829 (14.6)	15,626 (15.0 [14.8,15.2])	0.005	-2797	17.90
Lung	**Within 5 miles**	39,503 (27.0)	47,646 (27.3 [27.1,27.6])	0.04	-8143	17.09
	**5-10 miles**	31,018 (21.2)	36,689 (21.1 [20.9,21.3])	0.56	-5671	15.46
	**10-15 miles**	19,126 (13.1)	22,597 (13.0 [12.8,13.1])	0.88	-3471	15.36
	**15-35 miles**	33,031 (22.6)	38,436 (22.1 [21.9,22.3])	<.001	-5405	14.06
	**> 35 miles**	23,437 (16.0)	28,851 (16.6 [16.4,16.7])	<.001	-5414	18.76
Melanoma	**Within 5 miles**	9,429 (18.2)	14,354 (19.3 [19.0,19.6])	<.001	-4925	34.31
	**5-10 miles**	9,935 (19.2)	14,018 (18.8 [18.6,19.1])	0.28	-4083	29.13
	**10-15 miles**	7,572 (14.6)	10,974 (14.7 [14.5,15.0])	>.99	-3402	31.00
	**15-35 miles**	13,610 (26.3)	19,087 (25.7 [25.3,26.0])	0.008	-5477	28.69
	**> 35 miles**	11,286 (21.8)	15,976 (21.5 [21.2,21.8])	0.46	-4690	29.35
Stomach	**Within 5 miles**	4,392 (27.1)	5,261 (27.6 [26.9,28.2])	0.69	-869	16.53
	**5-10 miles**	3,661 (22.6)	4,096 (21.5 [20.9,22.1])	0.004	-435	10.62
	**10-15 miles**	2,104 (13.0)	2,461 (12.9 [12.4,13.4])	>.99	-357	14.52
	**15-35 miles**	3,311 (20.4)	3,854 (20.2 [19.6,20.8])	>.99	-543	14.10
	**> 35 miles**	2,760 (17.0)	3,401 (17.8 [17.3,18.4])	0.03	-641	18.85
Other	**Within 5 miles**	165,721 (23.9)	198,704 (23.7 [23.6,23.8])	0.007	-32983	16.60

© 2023 Janczewski LM et al. *JAMA Network Open*.

	**5-10 miles**	142,648 (20.5)	169,883 (20.3 [20.2,20.4])	<.001	-27235	16.03
	**10-15 miles**	93,862 (13.5)	112,496 (13.4 [13.4,13.5])	0.08	-18634	16.56
	**15-35 miles**	153,791 (22.2)	183,126 (21.9 [21.8,21.9])	<.001	-29335	16.02
	**> 35 miles**	138,130 (19.9)	173,806 (20.7 [20.7,20.8])	<.001	-35676	20.53

© 2023 Janczewski LM et al. *JAMA Network Open*.

eTable 3. Coordination of Care Received at Single Versus Multiple Institutions by Cancer Site in the Year 2020

Primary Site	Coordination of Care	Observed No. (%)	Expected No. (% [95% CI])	Corrected p-Value	Observed - Expected	% Decrease in 2020
**All Sites Combined**	**Multiple**	583,738 (47.5)	702,976 (47.7 [47.6,47.7])	<.001	-119238	16.96
	**Single**	592,763 (48.2)	693,483 (47.0 [46.9,47.1])	<.001	-100720	14.52
**Breast**	**Multiple**	137,994 (59.1)	161,106 (58.3 [58.1,58.5])	<.001	-23112	14.35
	**Single**	87,175 (37.4)	101,913 (36.9 [36.7,37.1])	<.001	-14738	14.46
**Colon**	**Multiple**	28,022 (43.1)	33,182 (43.2 [42.8,43.6])	>.99	-5160	15.55
	**Single**	34,493 (53.1)	40,486 (52.7 [52.4,53.1])	0.150	-5993	14.80
**Lung**	**Multiple**	60,849 (43.4)	73,132 (44.1 [43.9,44.4])	<.001	-12283	16.80
	**Single**	73,398 (52.3)	84,759 (51.2 [50.9,51.4])	<.001	-11361	13.40
**Melanoma**	**Multiple**	30,456 (58.0)	38,686 (56.4 [56.0,56.7])	<.001	-8230	21.27
	**Single**	19,960 (38.0)	26,226 (38.2 [37.8,38.6])	0.960	-6266	23.89
**Stomach**	**Multiple**	7,158 (54.0)	8,582 (54.5 [53.7,55.3])	0.710	-1424	16.59
	**Single**	5,471 (41.3)	6,266 (39.8 [39.0,40.6])	0.002	-795	12.68

© 2023 Janczewski LM et al. *JAMA Network Open*.

eTable 4. Utilization of Treatment in the Year 2020 Compared With Prior Years

Primary Site	Treatment Modality	Observed No. (%)	Expected No. (% [95% CI])	Corrected p-Value	Observed - Expected	% Decrease in 2020
All Sites Combined	Surgery	702,480 (57.1)	849,285 (57.6 [57.5,57.7])	<.001	-146805	17.29
	Chemotherapy	392,860 (31.9)	460,874 (31.2 [31.2,31.3])	<.001	-68014	14.76
	Radiation	362,556 (29.5)	443,036 (30.0 [29.9,30.1])	<.001	-80480	18.17
Breast	Surgery	209,363 (89.7)	244,109 (88.3 [88.2,88.4])	<.001	-34746	14.23
	Chemotherapy	72,833 (31.2)	84,474 (30.5 [30.4,30.7])	<.001	-11641	13.78
	Radiation	121,876 (52.2)	144,760 (52.3 [52.1,52.4])	>.99	-22884	15.81
Colon	Surgery	52,586 (80.9)	62,947 (82.0 [81.7,82.3])	<.001	-10361	16.46
	Chemotherapy	25,462 (39.2)	29,291 (38.3 [38.0,38.7])	<.001	-3829	13.07
	Radiation	2,229 (3.4)	2,739 (3.6 [3.4,3.7])	0.180	-510	18.63
Lung	Surgery	32,543 (23.2)	40,975 (24.6 [24.4,24.8])	<.001	-8432	20.58
	Chemotherapy	56,120 (40.0)	66,283 (40.2 [39.9,40.4])	0.60	-10163	15.33
	Radiation	57,677 (41.1)	69,800 (41.8 [41.6,42.1])	<.001	-12123	17.37
Melanoma	Surgery	49,191 (93.6)	62,666 (92.0 [91.8,92.2])	<.001	-13475	21.50
	Chemotherapy	801 (1.5)	1,876 (2.8 [2.6,2.9])	<.001	-1075	57.30
	Radiation	1,580 (3.0)	2,716 (4.0 [3.8,4.1])	<.001	-1136	41.82
Stomach	Surgery	4,939 (37.3)	6,362 (40.6 [39.8,41.3])	<.001	-1423	22.36
	Chemotherapy	7,845 (59.2)	9,090 (58.4 [57.6,59.1])	0.170	-1245	13.70
	Radiation	3,229 (24.4)	3,999 (25.5 [24.8,26.2])	0.008	-770	19.27

© 2023 Janczewski LM et al. *JAMA Network Open*.

eTable 5. Utilization of Treatment by Facility Type During the COVID-19 Pandemic

Primary Site	Facility Type	Observed No. (%)	Expected No. (% [95% CI])	Corrected p-Value	Observed - Expected	% Decrease in 2020	Decrease in Patients (n)
All Sites Combined	Academic/Research Program	449,529 (36.6)	554,622 (38.0 [38.0,38.1])	<.001	-105093	18.95	484
	Community Cancer Program	501,157 (40.8)	573,589 (39.3 [39.3,39.4])	<.001	-72432	12.63	99
	Integrated Network Program	276,752 (22.5)	317,579 (21.8 [21.7,21.8])	<.001	-40827	12.86	110
Breast	Academic/Research Program	72,590 (31.1)	87,484 (31.6 [31.5,31.8])	<.001	-14894	17.03	69
	Community Cancer Program	103,068 (44.2)	119,190 (43.1 [42.9,43.3])	<.001	-16122	13.53	22
	Integrated Network Program	57,079 (24.5)	66,295 (24.0 [23.8,24.1])	<.001	-9216	13.90	25
Colon	Academic/Research Program	18,285 (28.1)	22,370 (29.6 [29.2,29.9])	<.001	-4085	18.26	19
	Community Cancer Program	30,937 (47.6)	35,794 (47.3 [46.9,47.6])	<.001	-4857	13.57	7
	Integrated Network Program	15,654 (24.1)	17,500 (23.1 [22.8,23.4])	<.001	-1846	10.55	5
Lung	Academic/Research Program	42,312 (30.1)	53,153 (32.0 [31.8,32.2])	<.001	-10841	20.40	50
	Community Cancer Program	65,922 (47.0)	75,279 (45.3 [45.1,45.6])	<.001	-9357	12.43	13
	Integrated Network Program	31,881 (22.7)	36,857 (22.2 [22.0,22.4])	<.001	-4976	13.50	13
Stomach	Academic/Research Program	5,027 (37.9)	6,301 (40.5 [39.8,41.3])	<.001	-1274	20.22	6
	Community Cancer Program	5,256 (39.6)	5,856 (37.7 [36.9,38.4])	<.001	-600	10.24	1
	Integrated Network Program	2,952 (22.3)	3,300 (21.2 [20.6,21.9])	<.001	-348	10.56	1

© 2023 Janczewski LM et al. *JAMA Network Open*.

eFigure 2. Number of Hospitals per Facility Type in the NCDB

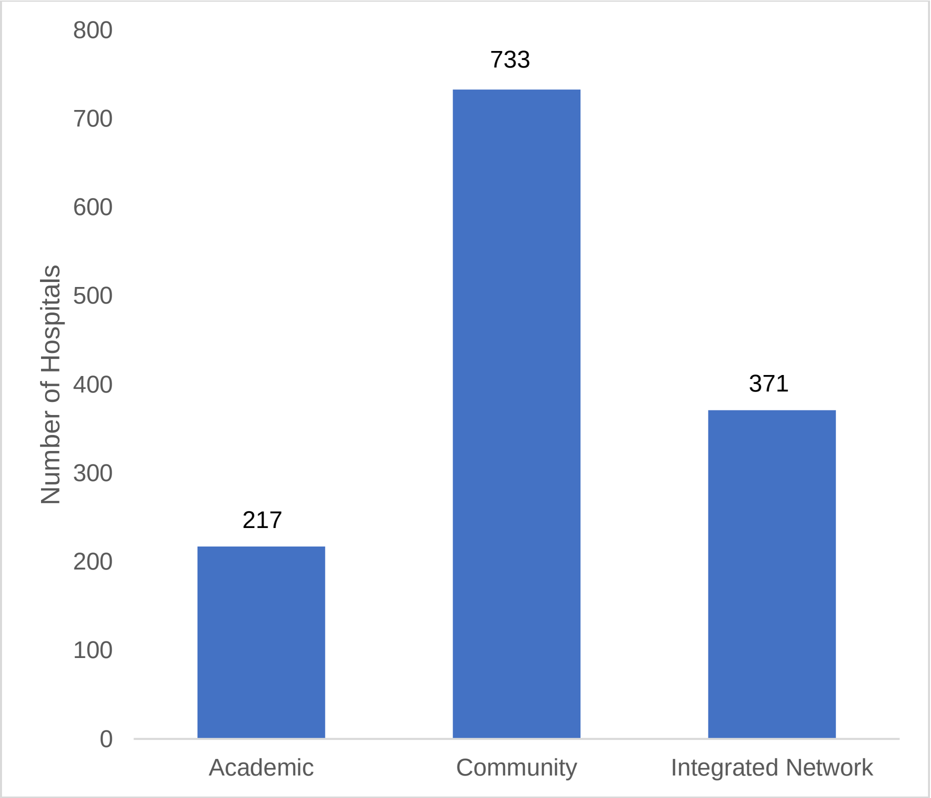

© 2023 Janczewski LM et al. *JAMA Network Open*.

eFigure 3. Utilization of Treatment in the Year 2020 Compared With Prior Years by Primary Site

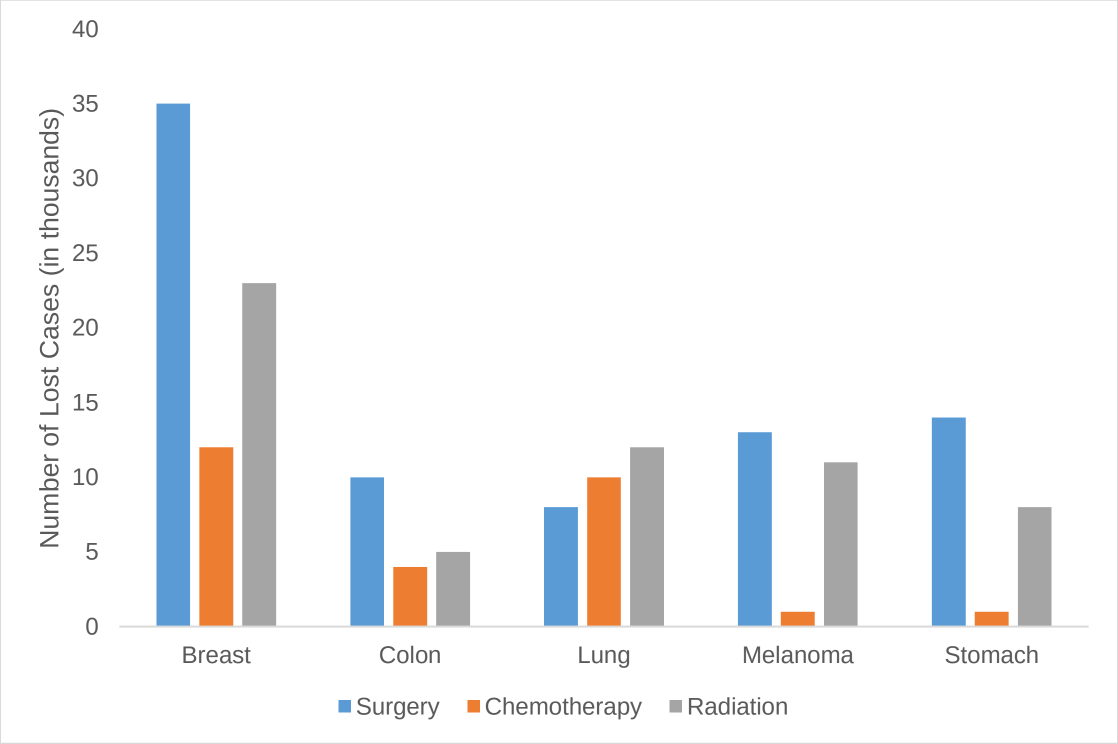

© 2023 Janczewski LM et al. *JAMA Network Open*.
