# Supplementary material for: Alterations in Cancer Treatment During the First Year of the COVID-19 Pandemic in the US
Source: JAMA Netw Open. 2023 Oct 30;6(10):e2340148. doi: 10.1001/jamanetworkopen.2023.40148 (PMC10616721; doi:10.1001/jamanetworkopen.2023.40148)
Supplement: Supplement 2. — Data Sharing Statement [file jamanetwopen-e2340148-s002.pdf]

Data Sharing Statement

Janczewski. Alterations in Cancer Treatment During the First Year of the COVID-19 Pandemic in the US. *JAMA Netw Open*. Published October 30, 2023.
doi:10.1001/jamanetworkopen.2023.40148

Data
Data available: No

Additional Information
Explanation for why data not available: This data came from the NCDB and is restricted in its use beyond the original sharing. The data can readily be obtained through the NCDB for eligible users.
